# *Gastrodia elata* Blume (Tianma): Hope for Brain Aging and Dementia

**DOI:** 10.1155/2020/8870148

**Published:** 2020-12-27

**Authors:** Klaus Heese

**Affiliations:** Graduate School of Biomedical Science and Engineering, Hanyang University, 222 Wangsimni-ro, Seongdong-gu, Seoul 133791, Republic of Korea

## Abstract

Since aging-related diseases, including dementia, represent major public health threats to our society, physician-scientists must develop innovative, interdisciplinary strategies to open new avenues for development of alternative therapies. One such novel approach may lie in traditional Chinese medicine (TCM). *Gastrodia elata* Blume (*G. elata*, tianma) is a TCM frequently used for treatment of cerebrocardiovascular diseases (CCVDs). Recent studies of *G. elata-*based treatment modalities, which have investigated its pharmacologically relevant activity, potential efficacy, and safety, have employed *G. elata* in well-characterized, aging-related disease models, with a focus on models of aging-related dementia, such as Alzheimer's disease (AD). Here, I examine results from previous studies of *G. elata,* as well as related herbal preparations and pure natural products, as prophylaxis and remedies for aging-related CCVDs and dementia. Concluding, data suggest that tianma treatment may be used as a promising complementary therapy for AD.

## 1. Introduction

Aging-related dementia, which is mediated by damage to brain cells induced by pathways, such as those underlying Alzheimer's disease (AD), cerebrocardiovascular diseases (CCVDs), and other neurodegenerative diseases (NDs), is causing great inquietude, anxiety, and discomposure in an aging society [1–7]. The World Health Organization (WHO) has recognized the imperative for globally coordinated research to combat dementia [8]. Much hope has been based on use of stem cell-based therapies; however, such approaches still have to overcome major challenges [9].

Thus, with dementia posing a health threat to elderly people, social awareness of healthy lifestyle choices that can prevent aging-related neuroinflammation and cognitive dysfunction has been attracting increasing attention. In particular, a healthy diet, exercise, and caloric restriction have been demonstrated to be preventive against new-onset AD and to effectively ameliorate the symptoms of AD [10, 11]. Familial (early-onset, younger than 65 years) AD is caused by genetic mutations [12–15]. However, the majority of AD cases (∼95%) is the sporadic non-inherited form, which is also referred to as late-onset (non-familial, sporadic) AD [14, 16]. Sporadic AD is likely caused by normal aging [16, 17] and its associated consequences, including oxidative stress and disturbance of protein homeostasis [13, 18–20].

Recently, many companies have stopped their AD-related clinical trials and minimized their investments in neurological studies [21]. Therefore, we need new approaches to open doors for alternative therapeutic strategies against aging-related NDs and dementia. In the past few years, alternative medicine has come into focus for the potential to provide new therapeutic measures for dementia [22–25]. Recent comparative proteomics research studies regarding AD-related TCM treatments revealed novel data that suggest that potential mechanisms of action of TCM for the prevention of AD pathogenesis involve improving the ubiquitin proteasome system (UPS, including chaperones and cochaperones (notably, heat shock proteins (HSPs) and FK506 binding proteins (FKBPs))) [20, 26]. Particularly, *G. elata* (tianma) received special attention and will therefore be discussed in more detail as follows [26].

## 2. *G. Elata* (Tianma) and NDs

*G. elata* (tianma) is a member of the Orchidaceae family and has its origin in East Asia. Its tuber has been used in TCM for centuries [26–30], and extracts of tianma or its active ingredients convey physiological- and health-promoting features, including antitumor, memory improving, and neuroprotective activities [30–33]. Particularly, this TCM has been widely used in Asia to treat dizziness, paralysis, epilepsy [34], and hypertension [35]. Tianma has also been used in this region to overcome cognitive deficits and prevent NDs [30, 36–41], including AD [42–46], vascular dementia (VD) [33, 41], and Parkinson's disease (PD) [47, 48], with gastrodin and 4-hydroxybenzyl alcohol among the primary active components [48–53].

## 3. Tianma Mobilizes the Cerebrocardiovascular System

It is common knowledge that heart health contributed to brain health. Connections between AD, VD, diabetes mellitus (type 2, T2DM), and CCVDs have been proposed based on the strong associations between cardiovascular risk factors and AD and VD, suggesting that these diseases share common characteristics [54–57]. The risk of developing aging-related AD, VD, and CCVDs appears to be increased with a wide range of conditions and lifestyle factors, including global failure of cellular energy metabolism, hypertension, dyslipidemia, hypercholesterolemia, lower physical activity, and poor diet [22, 56, 58–66].

### 3.1. Tianma Enhances Acetylcholine- (ACh-) Induced Vasorelaxation, A Measure of the Contractile Force and Elasticity of Aortic Vessels: Vasodilatory Proteomic Profile Changes in Aortic Tissue

**Figure 1:**
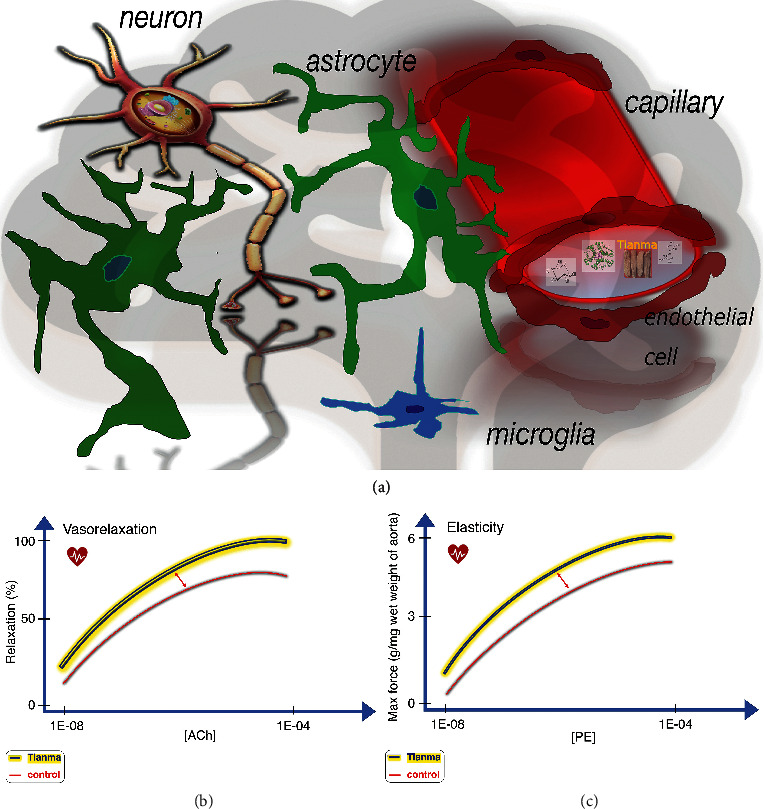
Schematic overview of tianma-mediated CCV-related activities. (a) With astrocytes serving as sensors and mediators between neural signal transmission and the vascular-dependent energy (glucose) supply, tianma can improve vascular activities and, upon uptake into the cerebrum, neuronal activities and survival and provide neuroprotection against ischemic strikes [33, 41]. (b) Quantitative data showing tianma-enhanced vasorelaxation. Elderly rats were treated with tianma for a period of three months (∼2.5 g/kg/day), after which their thoracic aortas were isolated. Dose-response analysis with increasing level of acetylcholine- (ACh-) induced relaxation in KCl (80 mM) or phenylephrine (PE, 10^−6^ M) precontracted isolated endothelium-intact arterial rings [67]. (c) Quantitative representation of tianma-increased vascular contractile force and elasticity. Dose-response comparison of maximum contractile force in response to increasing concentration of PE in endothelium-intact thoracic aortic rings (preincubated with 80 mM K^+^) in tianma-treated elderly rats and controls [67].

Blood vessel tonicity is regulated by vascular smooth muscle cells which modulate contraction and relaxation. Functional aortic tissue proteomic data have demonstrated that long-term treatment with small doses of tianma regulated blood vessel tonicity by mediating the expression of contractile proteins (e.g., actin alpha 2 (ACTA2)) and structural proteins (e.g., desmin (DES), microtubule-associated protein 4 (MAP4), PDZ, and LIM domain 1 (PDLIM1) and vinculin (VCL)), extracellular matrix proteins (ECM, e.g., elastin (ELN), fibulin 5 (FBLN5), and proline- and arginine-rich end leucine-rich repeat protein (PRELP)), and thrombotic proteins (e.g., annexin A2 (ANXA2)), thereby enhancing thoracic aortic contractile force and improving blood vessel elasticity (Figure 1) [67]. Moreover, elevated ANXA2 and reduced level of fatty acid binding protein 4 (FABP4) may prevent atherosclerosis and cardiovascular diseases [68, 69].

By inductive reasoning, tianma could likely prevent many CCVDs, such as headache, hypertension, atherosclerosis, and stroke, by facilitating vasodilatory effects that strengthen the arterial structure. Therefore, identification of all the bioactive ingredients in tianma could help facilitate its application as an efficient therapeutic herbal medicine for treatment of CCVDs by elucidating the mechanisms by which it ameliorates these abnormal cardiovascular responses [33, 41, 54, 67, 70, 71].

## 4. Tianma Improves Cognitive Function during Aging-Related Dementia

**Figure 2:**
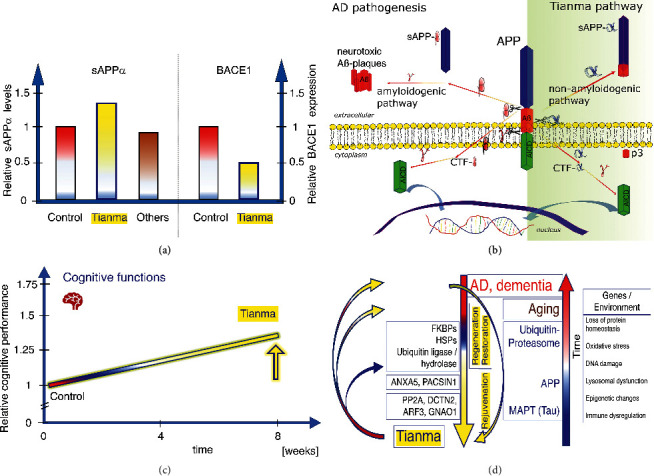
The effects of tianma on AD-related signaling and cognitive function during aging. (a) Qualitative data showing the effects of tianma and other herbs on APP processing. Estimation of soluble sAPP*α* level in cell culture supernatant (measured by enzyme-linked immunosorbent assay (ELISA)) and relative beta-secretase 1 (BACE1) expression (evaluated by Western blot analyses) of neuronal cells treated with tianma. Neurons were grown and treated with certain concentrations of tianma or other herbs for 24 h. Data are qualitatively represented as relative changes compared with controls. Only tianma-treated neurons showed significant increase of sAPP*α* level and significantly reduced BACE1 expression [44, 46]. (b) Schematic overview of the impact of tianma on APP processing. At the measured concentrations, tianma inhibits BACE1, promotes sAPP*α* production, suppresses amyloid beta peptide (A*β*) plaque formation, and reduces microtubule-associated protein tau (MAPT) phosphorylation, thereby fostering the nonamyloidogenic pathway [42, 44, 46]. (c) Qualitative data showing the effects of tianma on cognitive function. Tianma enhances memory, learning, and executive function in elderly rats during the Morris water maze, object recognition, and attention set shift tests [32, 41, 43, 46, 49]. (d) Schematic overview of the mechanisms underlying the effects of tianma on dementia. Tianma markedly improves cognitive abilities and protects against aging-related dementia, memory impairment, and neurodegeneration by restoring and rejuvenating cerebrovascular functions in elderly rats [30, 32, 33, 37, 40–44, 46, 49, 72].

Accumulating evidence indicates that tianma sharpens several cognitive functions, including memory and learning activities [30, 32, 40, 43, 49]. Moreover, neuroprotective and neuro-regenerative qualities have been attributed to tianma, particularly during aging and aging-related NDs, such as AD, PD, and VD [26, 30, 36, 38–44, 47, 72]. Specifically, pharmacologically relevant studies have demonstrated at the cellular and molecular levels that tianma could prevent AD by modulating proteolytic processing of amyloid beta precursor protein (APP), driving the nonamyloidogenic pathway (Figure 2) [41–44, 46].

## 5. Discussion

### 5.1. Aging and Dementia: Abnormal Protein Structures

In AD, accumulation of A*β* and hyperphosphorylated MAPT protein act as seeds for prion-like transmission of misfolded proteins to adjacent neurons, where misfolded MAPT further aggregates into neurofibrillary tangles (NFTs) [73–75]. The FKBPs act as a cochaperone in AD brains trying to prevent MAPT degradation by binding to MAPT and increasing its stability via interaction with the peptidylprolyl isomerase (PPIase) domain [76, 77]. However, downregulation of important E3-ligases (tripartite motif containing 32/37 (TRIM32/37)) and chaperone proteins, such as HSPs (e.g., HSP90), might impair hyperphosphorylated MAPT clearance [20, 37]. HSP90 and STUB1 (STIP1 (stress-induced phosphoprotein 1) homology and U-box containing protein 1, also known as carboxyl terminus of heat shock cognate 70- (HSC70-) interacting protein (CHIP)), target hyperphosphorylated MAPT  for proteasomal degradation. Hyperphosphorylated MAPT loses its physiological function for axonal transport, aggregates into NFTs, and causes neuron death. In addition, the impaired UPS (consisting of the 26S proteasome, ubiquitin ligases, and ubiquitin hydrolases) and compromised function of HSPs and FKBPs together impair the protein degradation pathway and promote pathophysiological conditions [20, 26, 37].

### 5.2. Interference Prevents Protein Misfolding during Aging and in NDs

The proposed pathomechanism underlying AD involves A*β* plaque formation, NFTs, and deregulation of chaperone proteins. Consequently, in AD brains, an impaired UPS system is thought to account for A*β* aggregation and hyperphosphorylated MAPT-mediated NFT formation, which is potentially furthered by an irregular APP intracellular domain (AICD) signaling pathway [20]. The various protein groups modulated by tianma treatment affect the UPS system, and active tianma ingredients also target molecular chaperones and cochaperones, such as HSPs and FKBP, and modulate the actions of protein phosphate PP2A. Together, these data open new avenues for future investigations into the prophylactic effects of tianma for aging-related dementia and NDs (Figure 2(d)) [20, 26].

## 6. Conclusion

The human brain, with its high-level cognitive functions, requires a large degree of flexibility and adaptability for appropriate learning and memory and is very vulnerable to cerebrovascular injuries, such as ischemia or stroke, which can cause NDs and dementia. Tianma has been shown in human clinical studies to be effective against VD [40], and various pre-clinical studies have demonstrated at the molecular and cellular levels its potential as an efficacious anti-aging elixir.

## Abbreviations

A*β*: Amyloid beta peptide
ACh: Acetylcholine
ACTA2: Actin alpha 2
AD: Alzheimer's disease
AICD: APP intracellular domain
ANXA2: Annexin A2
APP: Amyloid beta precursor protein
BACE1: Beta-secretase 1
CCVD: Cerebrocardiovascular disease
DES: Desmin
DM: Diabetes mellitus
ELISA: Enzyme-linked immunosorbent assay
ELN: Elastin
EOAD: Early-onset AD
FABP4: Fatty acid binding protein 4
FBLN5: Fibulin 5
FKBP: FK506 binding protein
*G. elata*, tianma: *Gastrodia elata* Blume
HSC70: Heat shock cognate 70
HSP: Heat shock protein
LOAD: Late-onset (nonfamilial, sporadic) AD
MAP4: Microtubule-associated protein 4
MAPT: Microtubule-associated protein tau
ND: Neurodegenerative disease
NFT: Neurofibrillary tangles
PD: Parkinson's disease
PDLIM1: PDZ and LIM domain 1
PDZ: Postsynaptic density protein (PSD95)
Dlg1: Drosophila disc large tumor suppressor
zo-1: zonula occludens-1 protein
LIM: Lin11, Isl-1, Mec-3
PE: Phenylephrine
PPIase: Peptidylprolyl isomerase
PRELP: Proline- and arginine-rich end leucine-rich repeat protein
STIP1: Stress-induced phosphoprotein 1
STUB1: STIP1 homology and U-box containing protein 1
TCM: Traditional Chinese medicine
TRIM: Tripartite motif containing
UPS: Ubiquitin proteasome system
VCL: Vinculin
VD: Vascular dementia
WHO: World Health Organization.
